# Use of self-gated radial cardiovascular magnetic resonance to detect and classify arrhythmias (atrial fibrillation and premature ventricular contraction)

**DOI:** 10.1186/s12968-016-0306-6

**Published:** 2016-11-25

**Authors:** Eve Piekarski, Teodora Chitiboi, Rebecca Ramb, Li Feng, Leon Axel

**Affiliations:** 1 Department of Radiology, The Center for Advanced Imaging Innovation and Research (CAI2R), New York University School of Medicine, 660 First Ave, New York, NY USA; 2 Department of Radiology, Bernard and Irene Schwartz Center for Biomedical Imaging, New York University School of Medicine, New York, NY USA; 3 Sackler Institute of Graduate Biomedical Sciences, New York University School of Medicine, New York, NY USA

**Keywords:** Arrhythmia, Premature ventricular contraction, PVC, Atrial fibrillation, AF, RR intervals, Self-gated CMR, XD-GRASP

## Abstract

**Background:**

Arrhythmia can significantly alter the image quality of cardiovascular magnetic resonance (CMR); automatic detection and sorting of the most frequent types of arrhythmias during the CMR acquisition could potentially improve image quality. New CMR techniques, such as non-Cartesian CMR, can allow self-gating: from cardiac motion-related signal changes, we can detect cardiac cycles without an electrocardiogram. We can further use this data to obtain a surrogate for RR intervals (valley intervals: VV). Our purpose was to evaluate the feasibility of an automated method for classification of non-arrhythmic (NA) (regular cycles) and arrhythmic patients (A) (irregular cycles), and for sorting of common arrhythmia patterns between atrial fibrillation (AF) and premature ventricular contraction (PVC), using the cardiac motion-related signal obtained during self-gated free-breathing radial cardiac cine CMR with compressed sensing reconstruction (XD-GRASP).

**Methods:**

One hundred eleven patients underwent cardiac XD-GRASP CMR between October 2015 and February 2016; 33 were included for retrospective analysis with the proposed method (6 AF, 8 PVC, 19 NA; by recent ECG). We analyzed the VV, using pooled statistics (histograms) and sequential analysis (Poincaré plots), including the median (medVV), the weighted mean (meanVV), the total number of VV values (VVval), and the total range (VVTR) and half range (VVHR) of the cumulative frequency distribution of VV, including the median to half range (medVV/VVHR) and the half range to total range (VVHR/VVTR) ratios. We designed a simple algorithm for using the VV results to differentiate A from NA, and AF from PVC.

**Results:**

Between NA and A, meanVV, VVval, VVTR, VVHR, medVV/VVHR and VVHR/VVTR ratios were significantly different (*p* values = 0.00014, 0.0027, 0.000028, 5×10^−9^, 0.002, respectively). Between AF and PVC, meanVV, VVval and medVV/VVHR ratio were significantly different (*p* values = 0.018, 0.007, 0.044, respectively). Using our algorithm, sensitivity, specificity, and accuracy were 93 %, 95 % and 94 % to discriminate between NA and A, and 83 %, 71 %, and 77 % to discriminate between AF and PVC, respectively; areas under the ROC curve were 0.93 and 0.89.

**Conclusions:**

Our study shows we can reliably detect arrhythmias and differentiate AF from PVC, using self-gated cardiac cine XD-GRASP CMR.

## Background

Arrhythmia can be a serious condition; it is relatively common in conditions such as chronic heart failure and it can contribute to their severity. However, some arrhythmic patterns, such as atrial fibrillation (AF) or premature ventricular contractions (PVCs), can occur without any symptoms; these arrhythmic patterns represent some of the most common types of arrhythmia. Arrhythmias with irregular cardiac cycles can also degrade image quality for patients undergoing some cardiac imaging methods, such as cardiovascular magnetic resonance (CMR) or cardiac computed tomography (CT), that rely on synchronization of the imaging with the cardiac cycle (“gating”). However, detection of the electrocardiogram (ECG) can be unreliable in CMR systems. Hence, it would be useful to be able to directly detect arrhythmia in patients undergoing CMR and to classify the detected arrhythmic patterns, as part of the imaging process.

Some methods of CMR, including recent approaches to non-Cartesian (particularly radial) sampled CMR with compressed sensing reconstruction, allow self-gating, i.e., cardiac and respiratory motion-related signals can be directly derived from the acquired imaging data itself [1–3]. We can detect cardiac cycles in this motion-related signal data, this signal being quasi-periodic. The end-systolic phases typically appear as relative minima (“valleys”) of the cardiac motion-related signal. Therefore, we can derive a surrogate measure for the RR interval from this information, as the interval between consecutive cardiac motion-related signal valleys (VV interval), without the need of ECG recording. Since we only consider the RR interval in this approach, some regular arrhythmic patterns cannot be detected or classified in this way (e.g., atrial flutter with a fixed block ratio). However, it is typically the irregular arrhythmias that are most likely to degrade the imaging in gated cardiac imaging methods. Therefore, we will focus here on the use of the self-gating-derived cardiac motion data for the detection of irregular arrhythmias and for classifying patients as non-arrhythmic (NA) (regular rhythm) or irregularly arrhythmic (A); the arrhythmia patients are further classified as patients with atrial fibrillation (AF) and patients with premature ventricular contractions (PVC), the most commonly encountered irregular arrhythmias [4]. An accurate classification of the arrhythmic vs. non arrhythmic beats in the A group, using self-gated radial CMR, could potentially allow for improved reconstruction of cardiac cine MR and could offer more information regarding the heart’s physiology during the imaging. The purpose of our study was to specifically evaluate the feasibility of using an automated cardiac cycle detection and classification method to: 1) separate NA from A patients, and 2) further classify the A patients into AF and PVC groups, using the cardiac motion-related signal obtained during self-gated free-breathing golden-angle radial cardiac cine CMR with compressed sensing reconstruction (called XD-GRASP [5]).

## Methods

### Study population

In our IRB-approved study, 111 of 136 consecutive patients undergoing a conventional CMR protocol in our center, between October 2015 and February 2016, underwent an additional acquisition of a 2D single-slice short-axis cine, using the XD-GRASP sequence, as a part of the technical development of the imaging method. Documentation of consent was waived for a retrospective analysis of these data. In AF and PVC groups, we included all of these patients who were referred to CMR in the specific context of AF or PVCs, or with AF or PVCs documented on conventional ECG monitoring done prior to the CMR. A total of 27 patients were referred to CMR in the context of arrhythmia: 8 with AF, 8 with PVCs, 1 with atrial flutter, 5 with supraventricular tachycardia, and 4 with unspecified arrhythmia. 1 patient referred for evaluation of abnormal anatomy of the right coronary artery also had AF, and 1 patient with cardiac lipoma was found to have frequent PVCs, on ECGs acquired prior to CMR examination. Of the PVC patients, none presented with bigeminy nor trigeminy. Three patients who had undergone electrical cardioversion prior to CMR, along with 1 with a history of paroxysmal AF, were excluded, as they had evidence of sinus rhythm and no sign of arrhythmia at the time of the CMR examination. A total of 6 and 8 patients were thus included in the final AF and PVC groups for further analysis, respectively.

In the NA control group, we included patients who did not present with any personal nor familial history of arrhythmia, who had all had an ECG study acquired less than 3 months prior to the CMR showing sinus rhythm without conduction abnormalities. We excluded patients who presented with acute or subacute ischemic cardiomyopathy or myocarditis (conditions known to be prone to arrhythmia in the acute and subacute phases). A total of 19 patients were thus included in the NA group.

Therefore, a total of 33 patients were included in our study: 14 in the A group (6 in the AF group, 8 in the PVC group) and 19 in the control/NA group. A majority of patients were male (23 patients, 70 %); the average age was 42 years old for the NA group, and 60 years old in the A group (68 years old for the PVC group and 50 years old for the AF group). Clinical characteristics of the different groups are presented in Table 1.

**Table 1:** Characteristics of the population

		NA group	A group			Total
			All	PVC group	AF group	
Number of patients		19	14	8	6	33
Sex	Male	12/63 %	11/78.5 %	7/87.5 %	4/67 %	23/70 %
	Female	7/37 %	3/21.5 %	1/12.5 %	2/33 %	10/30 %
Age	Mean	42	60	68	50	50
	Median	42	70	72	50	52
	Max	73	80	80	73	80
	Min	15	17	52	17	15
Indications for MRI						
Arrhythmia		0	12	7	5	12
Cardiac Mass		0	1	1 PVC on ECG	0	1
Congenital CM		5	1	0	1 AF on ECG	6
Hypertrophic/dilated/overload CM		9	0	0	0	9
Duchenne CM		2	0	0	0	2
Pericardial disease		3	0	0	0	3

### CMR acquisition and cardiac motion detection

All patients underwent free-breathing XD-GRASP [5] for acquisition of a single-slice mid-ventricular short axis (SAX) cine. XD-GRASP uses continuous non-Cartesian sampling of the CMR k-space, using radial sampling [6, 7] with a golden-angle ratio sampling scheme (angular increment of ~111.25° for each consecutive spoke of samples), allowing us to sample k-space efficiently, using compressed sensing image reconstruction [8–13] (using a total variation constraint [14, 15] as a sparsifying transform, after a regridding operation). XD-GRASP (eXtra Dimension-Golden Angle Radial Sparse Parallel) combines the motion-robustness and self-navigation properties of radial imaging with the acceleration capabilities of compressed sensing. In particular, since each spoke traverses the center of k-space, we are able to extract both cardiac and respiratory motion signals, which are quasi-periodic, from the serial data at the center of k-space (which reflects the total image intensity) [2, 16, 17], and to reconstruct the image data after binning it into different cardiac and respiratory motion states. Since cardiac and respiratory motions are known to have different frequency ranges (0.6-2 Hz versus 0.1-0.4 Hz, respectively), we can separate and extract each motion component from the detected signals, particularly using the signal from receiver coil elements located over the heart and diaphragm, respectively. Instead of removing or correcting motion in the image reconstruction, the extra motion-state dimensions (e.g., cardiac and respiratory motion dimensions) are then explicitly jointly reconstructed; this can improve image quality and may also offer new insights into cardiorespiratory physiology [3, 18].

Imaging was performed during free-breathing in a whole-body 1.5T scanner (Avanto, Siemens AG, Erlangen, Germany) without external triggering or gating, using a 2D radial steady-state free precession (SSFP) sequence with golden-angle acquisition scheme. One short-axis (SAX) slice was acquired in each subject, with the following imaging parameters: TR/TE = 2.8/1.4 ms, FOV =320 × 320 mm^2^, number of readout points in each spoke = 128, spatial resolution = 2 × 2 mm^2^, slice thickness = 8 mm, acquisition time for each patient = 23 s.

### RR interval surrogate

Some prior studies have already used RR intervals obtained from ECG recordings [19, 20] or other types of cardiac recording [21], in order to detect and sort arrhythmias. In our study, we used a surrogate measure for RR intervals, as described below, using a custom program developed in MATLAB (Natick, MA, USA). RR intervals have been shown to be more robust to noise than p-wave-based algorithms [22]. After detection and filtering of the cardiac motion signal, we obtain a quasi-periodic signal. The filtering further uses threshold values of ratios of surrounding maxima to the detected minima to exclude spurious minima. With the automated detection of relative minima in the signal, we then extract the end-systole phases (seen as “valleys” or minima of the cardiac motion signal). End-systole intervals can be used as a surrogate for RR intervals; we will call the consecutive end-systole times, detected as signal valleys during the data acquisition, “V_i_”. The VV interval is defined as the time between 2 consecutive valleys (for example, VV_i_ equals the difference between the time of one valley, V_i_, and the time of the next valley, V_i+1_); this is an integer multiple of the TR time. For each patient, we analyzed the median (medVV), the weighted mean (meanVV, mean weighted by the number of VV values), the total number of VV values (VVval), the total range (VVTR) and half range (VVHR, i.e., second and third quartiles) of the cumulative frequency distribution of VV, and calculated the median to half range (medVV/VVHR) and half range to total range ratios (VVHR/VVTR).

The total number of VV values (VVval), the total range (VVTR) and the half range (VVHR) of the cumulative frequency distribution of VV, all provide information on the distribution of the VV values, including information about potential gaps (for example, a high total range with a limited VVval indicates the potential presence of gaps, as we could expect in PVC patients, as opposed to AF patients).

Since our data acquisition time during imaging is short (23 s), we detected only a limited number of valleys for each patient (16 to 36 after filtering), depending on the heart rate. Therefore, the commonly used rhythm classification methods, based on RR intervals using long runs of ECG signals, are not useful here. This limits us to relatively simple approaches to the rhythm analysis, as opposed to studies using more complex classifiers [23–25]. We plotted histograms of the VV values and Poincaré plots of consecutive VV values, in order to visually assess potential differences between NA and A groups, and differences between the AF and PVC groups within the A patient group [26, 27]. Poincaré plots have been previously used to analyze heart-rate variability [28], plotting each RR interval as a function of the preceding RR interval.

### Arrhythmia detection and sorting algorithm

We sought to develop a simple automated algorithm, using data derived from the self-gating cardiac motion-related signal, which could help differentiate non-arrhythmic from arrhythmic patients, and AF from PVC patients within the arrhythmic group. This approach can be referred to as a knowledge-based method [19]. We designed an algorithm to discriminate between NA and A patients, and between AF and PVC patients, using the VV intervals. We chose our classifiers according to the results of the comparison between A and NA groups, and between AF and PVC groups. To determine the appropriate threshold values for each classifier, we used the Receiver Operating Characteristic (ROC) curves. We chose our classifiers according to our comparison analysis of the NA and A group and of the AF and PVC group. We desired that the chosen classifiers would be able to discriminate between the corresponding 2 groups, and they were chosen according to their simplicity, and potential reproducibility (e.g., as VVTR, TotValVV and meanVV are all also related to the heart rate, we chose VVHR as a measure of the width of the distribution of VV values, without any change in our algorithm performance). The thresholds used for each classifier were determined using ROC analysis, as described below in the Results section.

The decision tree developed to discriminate between A and NA patients, and between AF and PVC patients, is presented in the Results section.

### Statistical analysis

To compare the A and NA groups, and the AF and PVC groups, we used Student’s *t* test regarding analysis of medVV, meanVV, VVval, VVTR, VVHR and medVV/VVHR ratio; we also used analysis of variance (ANOVA) and binary regression. Finally, we tested our arrhythmia diagnosis algorithm using different parameter values to assess for sensitivity, specificity, and accuracy regarding the classification of A vs. NA patients, and of PVC vs. AF patients in the A group. From these data, we obtained ROC curves and corresponding areas under the curve (AUC). Finally, to compare AF and PVC groups, we used a Gaussian mixture model analysis (MATLAB, Natick, MA, USA). A mixture model corresponds to the mixture distribution representing the probability distribution of observations in the overall population, making statistical inferences about the properties of sub-populations within the overall population, given only observations on the pooled population. A Gaussian mixture model evaluates the mean of the analyzed component (mu) and its covariance (sigma). Then it evaluates the quality of the model according to different function parameters : negative log likelihood function (NLLH), Akaike information criterion (AIC) and Bayesian information criterion (BIC). We chose to use Gaussian Mixture Models in order to evaluate if our initial hypothesis, that the VV intervals in the PVC group would be more likely to follow a Gaussian distribution with additional sub-group peaks (corresponding to the PVC beats) than the AF group, which would be more likely to follow a less organized distribution, was confirmed. We compared both results from the model (mu and sigma, mean and covariance) and evaluation of the model (NLLH, AIC and BIC).

## Results

### Cardiac motion detection and VV interval analysis

**Fig. 1:**
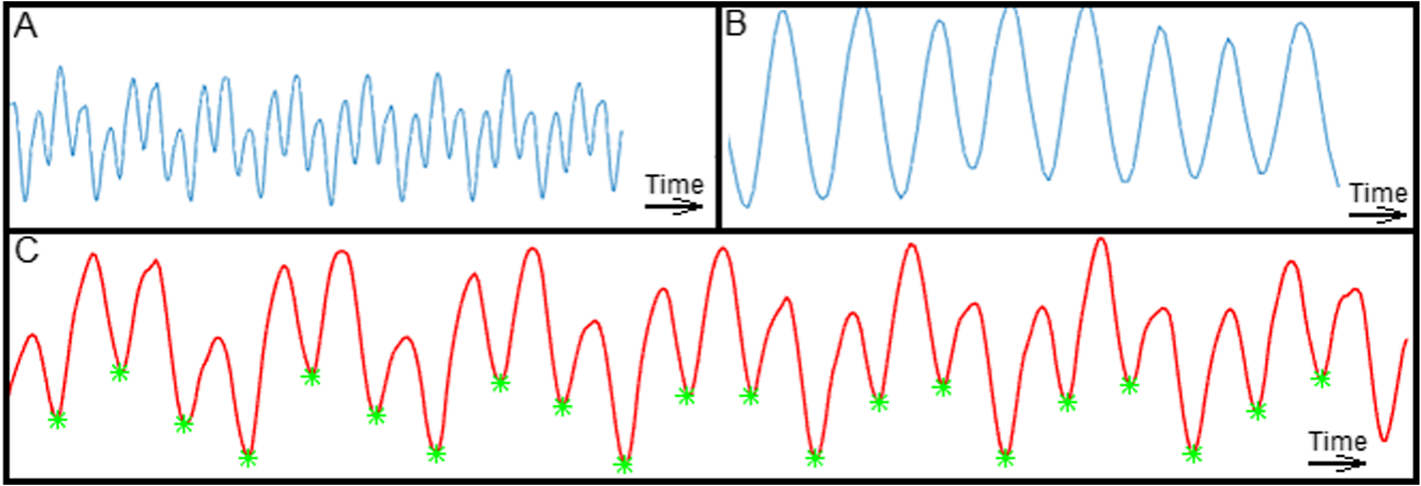
Example of respiratory modulation of cardiac motion-related signal. We can see that the joint motion-related signal (Image **a**) is modulated by the respiratory motion signal (Image **b**). However, valleys of the cardiac signal are still correctly detected (Image **c**, green stars) after the filtering step

**Fig. 2:**
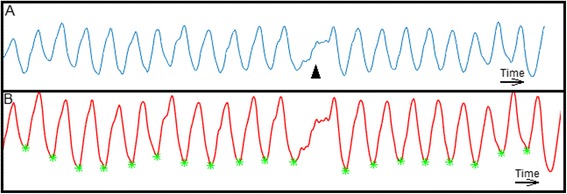
Example of loss of detection of a PVC beat after filtering step. We can qualitatively see (Image **a**) that the patient had a PVC beat (black arrow head). However, after the filtering step (Image **b**), the PVC beat was not automatically detected (detected valleys = green stars)

The 2D single-slice free-breathing XD-GRASP sequence allowed us to detect a reasonable-appearing cardiac motion-related signal in all patients. Some patients (12 in total: 2 in the PVC group and 10 in the NA group) showed appreciable modulation of the cardiac motion signal by the superimposed respiratory motion signal, but the cardiac motion-related valleys were still reliably detected (Fig. 1). Although our filtering step allowed us to discard irrelevant minima, corresponding to noise and contaminating the data, it was sensitive to the choice of a specific threshold value (which was empirically chosen), since it led to a loss of reliability of valley detection for some of our arrhythmia patients (in total: 2 of the 8 patients in the PVC group (Fig. 2)). Although some of the arrhythmic PVC beats were not recorded as a distinct valley, the following beat could be specifically detected as a post-extrasystolic beat, since the VV was longer than expected, in the context of otherwise regular beats.

Between NA and A groups, we found significant differences regarding meanVV, VVval, VVTR, VVHR, medVV/VVHR and VVHR/VVTR ratios (*p* values = 0.00014, 0.0027, 0.000028, 5x10^−9^, 0.002 respectively). Between AF and PVC groups, meanVV, VVval and medVV/VVHR ratio were significantly different (*p* values = 0.018, 0.007, 0.044, respectively). VV analyses are summarized in Tables 2 and 3.

**Table 2:** Results of VV data analysis: comparison between NA and A groups

	NA		A		*p* value
	Average	SD	Average	SD	
TotValVV	4.68	1.73	8.57	3.86	**0.0027**
meanVV (seconds)	5.05	2.26	2.48	0.92	**0.00014**
VVHR (seconds)	0.06	0.04	0.40	0.36	**0.0036**
VVTR (seconds)	0.22	0.09	1.11	0.53	**0.000028**
medVV/VVHR	16.59	5.68	4.10	2.63	**5×10** ^**−9**^
VVHR/VVTR	0.58	0.14	0.46	0.05	**0.002**

All *p* values presented in bold are statistically significant, i.e. *p* < 0.05

**Table 3:** Results of VV data analysis: comparison between AF and PVC groups

	AF		PVC		*p* value
	Average	SD	Average	SD	
TotValVV	11.67	2.25	7.63	2.26	**0.0069**
meanVV (seconds)	1.88	0.38	2.94	0.96	**0.018**
VVHR (seconds)	0.43	0.17	0.38	0.47	0.78 NS
VVTR (seconds)	0.93	0.19	1.24	0.67	0.26 NS
medVV/VVHR	2.58	1.74	5.25	2.69	**0.044**
VVHR/VVTR	0.45	0.04	0.46	0.06	0.81 NS

*NS* = not statistically significant

All *p* values presented in bold are statistically significant, i.e. *p* < 0.05

Variance was statistically different (ANOVA) between the groups (significance F = 2 × 10^−8^ between NA and A, 0.041 between AF and PVC). Binary regression found significant differences between NA and A groups, regarding VVTR and medVV/VVHR ratio (*p* values = 0.0047 and 0.0065, respectively).

Finally, Gaussian mixture model analysis showed a significant difference regarding the parameters evaluating the quality of the model: NLLH, AIC and BIC (*p* = 0.04 with one component analysis and *p* = 0.03 with two components analysis, respectively), therefore showing a better fit of the model with PVC rather than with AF patients (NLLH, AIC and BIC lower for the PVC group). No significant difference was found regarding the results of the model (mu, sigma). This confirmed our initial hypothesis that the PVC patients would be more likely to follow a mixture of Gaussian distributions (non-arrhythmic beats following a Gaussian distribution, with added peaks of PVC beats and beats following PVCs with a compensatory pause) than the AF patients (corresponding to a more disorganized distribution, with a wide variation of the VV). All results from Gaussian mixture model analysis are shown in Table 4.

**Table 4:** Results of Gaussian mixture model analysis: comparison between AF and PVC groups

	AF		PVC		*p* value
	Average	SD	Average	SD	
One component analysis					
Mu	19.9	3.7	24.6	9	0.21 NS
Sigma	15.8	7.7	21.6	24.2	0.54 NS
NLLH	128.5	24.7	96.8	26.2	**0.04**
AIC	267	49.3	203.6	52.4	**0.04**
BIC	272	50.3	207	52.8	**0.04**
Two components analysis					
Mu	8.3	7.8	19.2	18.8	0.17 NS
Sigma	6.4	4.5	19.2	21.4	0.14 NS
NLLH	116.1	24.7	84.1	22	**0.03**
AIC	254.2	49.3	190.2	44	**0.03**
BIC	265.4	51.5	199.1	47.2	**0.03**

All *p* values presented in bold are statistically significant, i.e. *p* < 0.05

### Histograms and Poincaré plots of VV intervals

**Fig. 3:**
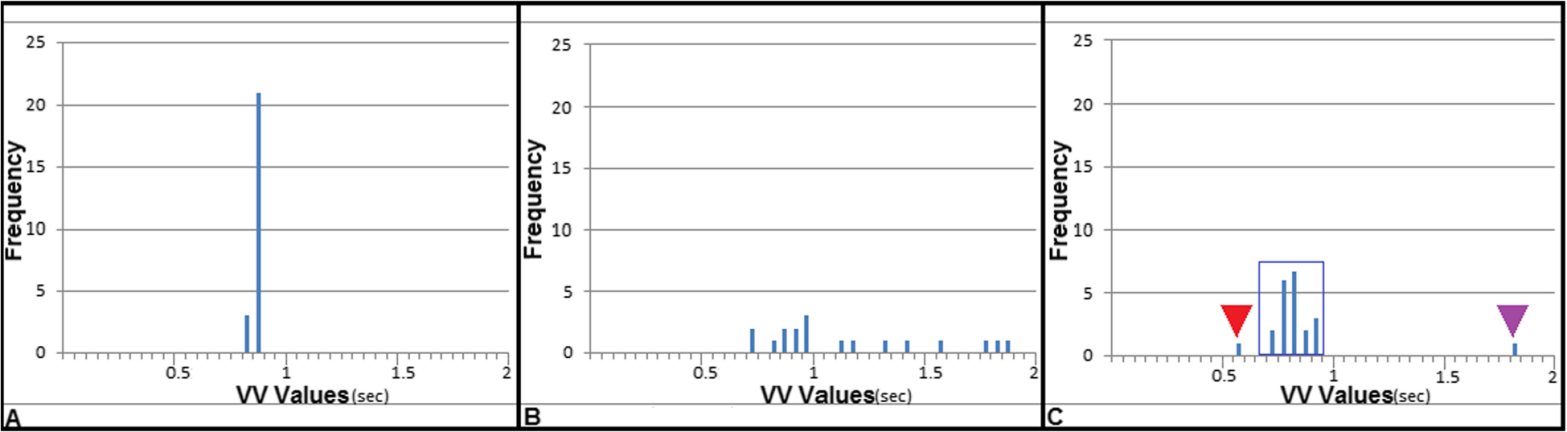
Representative histograms of VV values in: (Image **a**) a patient in normal sinus rhythm (NA group), (Image **b**) an AF patient and (Image **c**) a patient with isolated PVC. For the NA patient, the histogram demonstrates a relatively narrow Gaussian-like distribution, whereas for the AF patient, the histogram demonstrates a broader distribution of VV intervals. For the PVC patient, the histogram shows a distribution with 3 distinct populations (blue rectangle = regular “normal” beats, red arrow head = ectopic PVC beat and purple arrow head = delayed beat following compensatory pause after PVC)

**Fig. 4:**
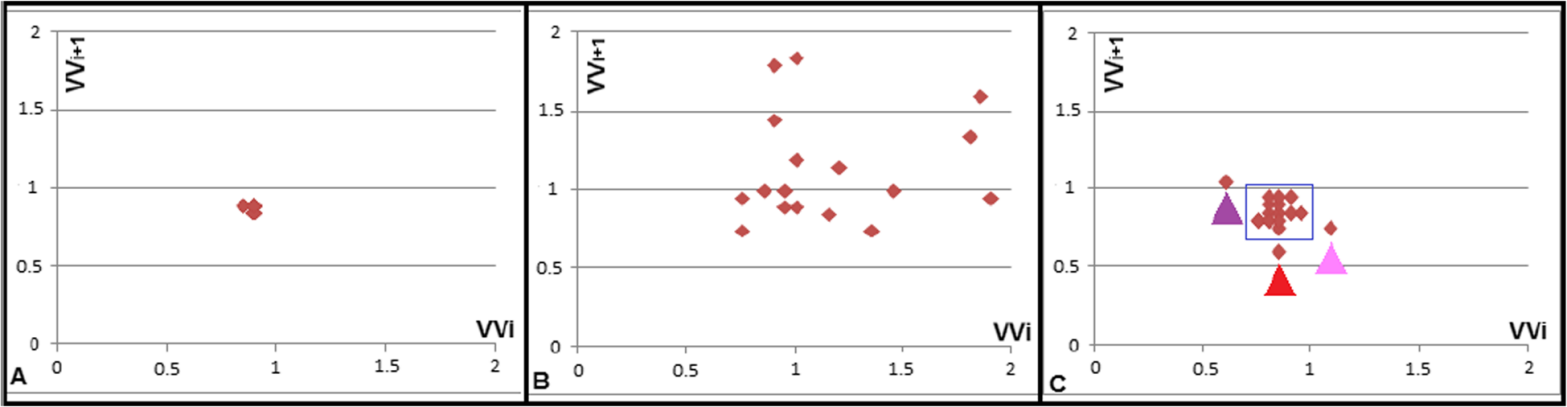
Representative Poincaré plots of (Image **a**) a patient in normal sinus rhythm (NA group), (Image **b**) an AF patient and (Image **c**) a patient with isolated PVC. For the NA patient, the Poincaré plot shows a narrow distribution with a single cluster; whereas for the AF patient, the Poincaré plot shows a broad, unorganized distribution. For the PVC patient, the Poincaré plot shows a distribution with 3 distinct populations (red arrow head = ectopic PVC beat, i.e., short beat following regular beat, purple arrow head = delayed beat following compensatory pause after PVC, i.e., long beat following short beat, and pink arrow head = recovery of normal sinus rhythm, i.e., regular beat following long beat), in addition to the regular “normal” beats (blue rectangle). VV_i_ and VV_i+1_ are expressed in seconds

For NA patients, we observed a relatively sharply peaked distribution of VV intervals in histograms and only one relatively tightly grouped population in the Poincaré plots (Figs. 3 and 4), corresponding to the known relatively small normal variability of heart rate in healthy subjects [29, 30]. AF patients presented a broader range of distribution in histograms and a disorganized distribution in Poincaré plots (Figs. 3 and 4), whereas histograms and Poincaré plots of PVC patients qualitatively showed 3 distinct populations of VV intervals: 1) “normal” sinus beats, 2) premature beats and 3) delayed beats, i.e., otherwise normal beats following a “compensatory pause” after a PVC (Figs. 3 and 4). The data were too limited in number to warrant formal statistical analysis of the plots.

### Arrhythmia detection and sorting algorithm

**Fig. 5:**
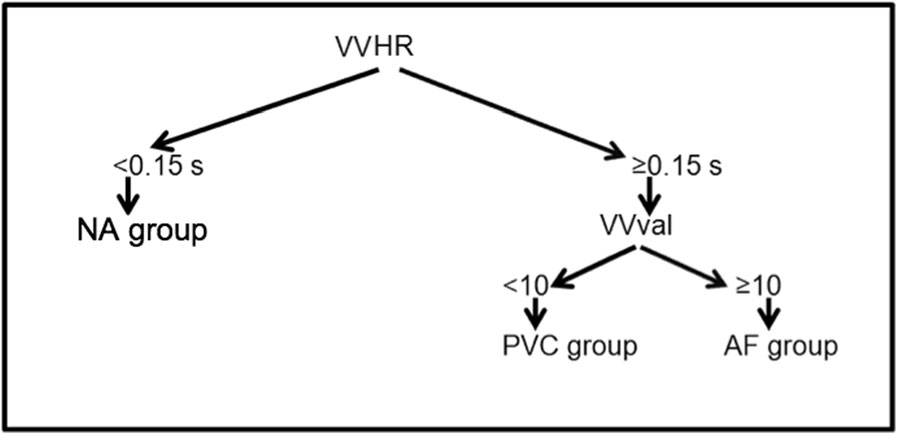
Arrhythmia detection and classification algorithm, as described in the text

The VV distribution-based arrhythmia detection and sorting algorithm that we have chosen to evaluate uses 2 steps (Fig. 5). First, if the half range of the cumulative frequency distribution of VV (VVHR) is strictly lower than 0.15 s, then the patient can be sorted into the NA group, as there is a sufficiently narrow range of VV values. Secondly, if VVHR is higher than or equal to 0.15 s, then we analyze the total number of VV values (VVval): if VVval is strictly lower than 10 (for our imaging parameters), then the patient can be sorted into the PVC group; on the contrary, if VVal is higher than or equal to 10, the patient can be sorted into the AF group.

We chose VVHR as the first classifier because it was able to discriminate strongly between the NA and A groups (mean VVHR =0.06 in NA group vs. 0.4 in A group, *p* < 0.005) while being an easy-to-determine parameter, being less dependent on the heart rate than meanVV or VVTR. We chose the total number of VV values as the second classifier because it was able to discriminate between the AF and PVC groups (mean TotValVV = 11.67 for the AF group vs. 7.63 for the PVC group, *p* < 0.01).

**Fig. 6:**
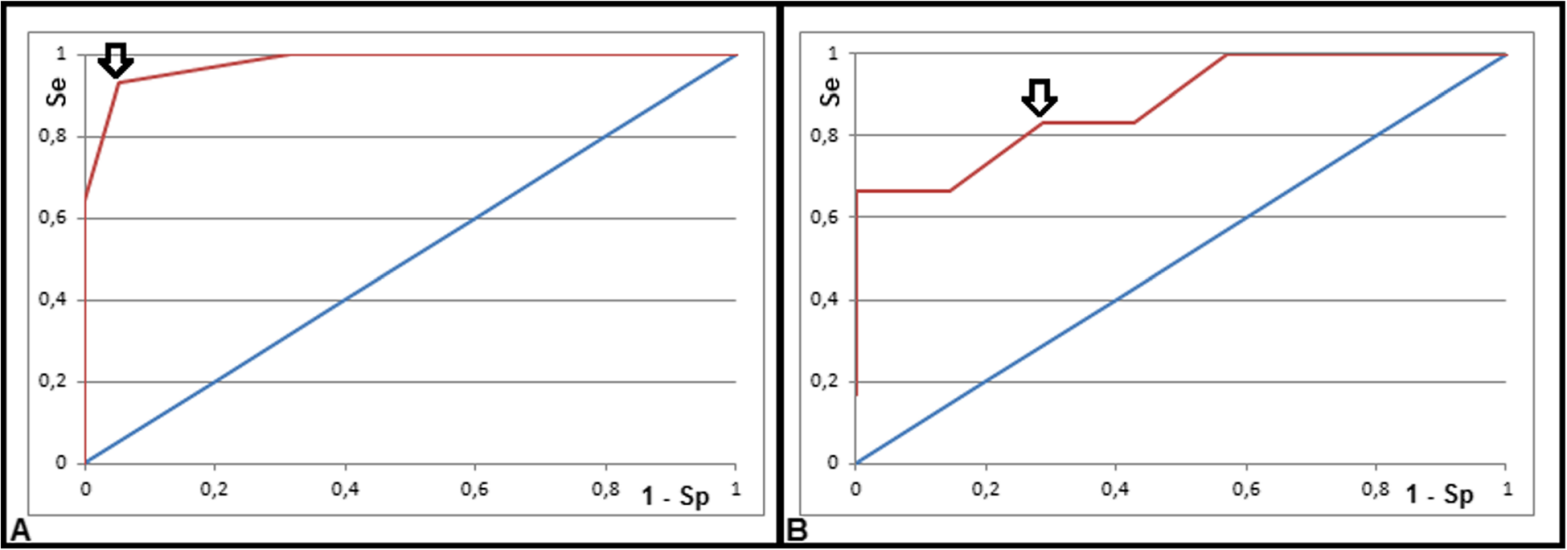
ROC curves of arrhythmia classification algorithm performance for classification of NA and A patients (Image **a**) (red curve; blue curve is line of identity for comparison) and for classification of AF and PVC patients (Image **b**). A black arrow shows the performance for the chosen parameter thresholds for VVHR and VVval on the **a** and **b** curves, respectively

We tested the results of using different threshold values for each classifier, and used the results of the ROC curve analysis to determine the optimal values for the thresholds (Fig. 6). Choosing a threshold value for VVHR strictly lower than 0.15 s to discriminate between NA and A patients, we obtained sensitivity, specificity, and accuracy of: 93, 95 and 94 %, respectively (1 false negative patient, i.e., an arrhythmic patient not detected by our algorithm). The area under the curve for this ROC curve was 0.93 (Fig. 6). Choosing a threshold value for VVval higher than or equal to 10 to discriminate between AF and PVC arrhythmia patients, we obtained sensitivity, specificity, and accuracy of: 83, 71 and 77 %, respectively (we only included in this analysis the patients detected as the A group by the first step of the algorithm). The area under the ROC curve was 0.89 (Fig. 6).

## Discussion

Our study found that the use of cardiac motion detection, as part of XD-GRASP cardiac cine CMR, also allowed for the calculation of an RR interval surrogate (through detection of the minima of cardiac motion-related signal, “valleys”, corresponding to end-systolic phases of the cardiac cycle, and calculation of the intervals between these valleys, VV) and that these data could help to automatically detect and discriminate between NA and A groups, and between AF and PVC groups within A. We found significant differences between these different groups, in a small sample of retrospectively analyzed patients, using simple variables extracted directly from the acquired CMR signal data itself, with the use of the self-gating properties of radial imaging.

No prior study has previously shown the feasibility of such cardiac rhythm classification based on the use of the CMR signal alone in patients. One prior study, on porcine hearts, studied arrhythmias with CMR, using Current Density Imaging and Diffusion Tensor Imaging [31]. Another study used compressed sensing to separate and analyze fetal-ECG signal from maternal abdominal ECG recordings [32].

Most prior studies concerning arrhythmia detection and classification have been based on relatively long duration ECG recordings, and have used a large number of heartbeats (e.g., thousands [33]). Although we acquired data for only a short time (23 s), we still were able to observe clear differences between NA and A patients, and between AF and PVC patients. Therefore, the use of VV intervals represents a potentially robust tool to automatically detect and sort arrhythmias in patients undergoing CMR. It is well known that intra-magnet ECG recordings can be significantly altered by the magnetic field; our use of a cardiac motion-related signal derived from the data itself, using the self-navigation properties of radial imaging, has allowed us to avoid this problem, although the use of VV values as a surrogate for RR values has other potential problems, such as a less sharp trough in the MR signal than the peak in the usual ECG signal QRS complex, thus potentially leading to more jitter in the derived timing.

While we have here focused on the use of the self-gating signal for the detection and classification of arrhythmias in patients undergoing CMR, the same arrhythmia analysis and classification principles could also be applied to the classification of patients undergoing conventional CMR, using the conventional monitoring ECG signal provided in the CMR system. In particular, patients with regular heart rates would be expected to have a relatively narrow range of RR values, while patients with atrial fibrillation would be expected to have a broader relative distribution of RR values than patients with PVCs. Similar findings for the RR intervals would be expected for patients undergoing other kinds of cardiac-gated imaging.

The most important aspect of this approach to cardiac motion-related signal analysis in arrhythmia is its potential for the improvement of overall image quality for arrhythmic patients undergoing CMR. In the current usual approach for CMR of arrhythmic patients with ECG monitoring, data are not acquired (or rejected) during heart beats that have RR intervals that differ significantly from the rest of the heart beats (“arrhythmia rejection”). This can lead to a subsequent loss of information and data on the rejected beats, impaired image quality if the rejection process is not fully reliable, and/or longer acquisition time. Many of the routine clinical CMR patients included in our retrospective analysis had significant image degradation due to arrhythmias. In particular, in the setting of atrial fibrillation, there are generally no truly regular beats, so that no simple rejection process can provide a set of fully consistent data; the more stringent the bounds of RR values for data acceptance are, the longer the data acquisition process will take. Hence, our new approach could help improve the image quality, by providing a means for automatically sorting and classifying abnormal beats, thus potentially allowing us to *a posteriori* choose a sliding temporal window of an optimal width for the retrospective image reconstruction and to handle different kinds of beats differently. Furthermore we can potentially use this information to enable reconstruction of images along an additional corresponding “dimension” related to the arrhythmia, which could allow us to study the effects of the arrhythmia on the cardiac contraction. In particular, while we have here focused on the overall classification of the cardiac rhythms of the patients from the self-gating cardiac signal, this analysis approach also provides an automated method for beat-by-beat classification of individual heart beats. Thus, we can potentially choose to separately reconstruct images of the ectopic beats, using compressed sensing methods to reconstruct the relatively “sparse” differences between the normal and ectopic beats, even with relatively few ectopic beats detected among the normal beats, and thus potentially provide us access to new kinds of information about the heart. This would allow the reconstruction of images of both normal and abnormal beats, as has been previously demonstrated with the XD-GRASP method [5] (without the use of this more automated approach). This could further improve the quality of the reconstructed data, without the loss of information entailed in the simple rejection of data from abnormal beats, as is used in conventional CMR. This approach could potentially improve image quality not only for CMR, but also for cardiac CT and other cardiac-gated imaging methods. In addition, this could potentially lead to new information on the physiology and pathophysiology of the heart, including its response to changing pre-load with varying filling times and the associated volume adaptation to arrhythmic patterns, on a beat-to-beat basis. Such reconstruction would necessitate acquiring imaging data on enough arrhythmic beats to allow them to be reconstructed separately. However, the problems of reconstructing images in the presence of the associated data sparseness expected along this new “preceding RR interval” dimension could potentially be resolved by using adequate compressed sensing methods, therefore limiting the potential blurring or other image artifacts otherwise likely to be associated with such highly undersampled data. Further studies are needed to evaluate the feasibility of those new types of cardiac image reconstruction.

This study has some limitations. First, since it was an exploratory retrospective study, concerning detection and classification of arrhythmia effects in a set of previously acquired radial imaging data, using self-gating imaging methods, we didn’t have available any recordings of simultaneous ECG signals in the CMR magnet during the image data acquisition (this is not part of routine clinical CMR). We have tried to compensate for that limitation by being fairly strict regarding our inclusion and exclusion criteria for the patients we analyzed, in order to make sure we initially included patients in the correct groups. However, these results cannot yet be generalized to a broader clinical population undergoing CMR, and further studies using both self-gated acquisitions and simultaneous ECG recordings will be needed to better define the patient characteristics in different rhythm states, and their effect on the success of these classification methods. A second important limitation is the inclusion of only a relatively small number of patients in all the groups, because of the limited initial pool of imaging data available for analysis, and the need to ensure that they were correctly classified into groups. Since we are presenting results on only a limited sample of patients, the potential generalizability of the results is limited, and studies with larger numbers of subjects and more statistical power will be needed to confirm our initial results. Third, if our detection of the cardiac motion signal is contaminated by noise, even though we use filtering, we might inadvertently mistake a local spurious minimum as a real valley, or fail to detect a real valley, therefore potentially incorrectly detecting arrhythmia. The presence of AF may also make the valley detection less reliable, as the irregular motion signal cannot be as easily filtered as the normal case. For 2 of the PVC patients, the filtering step resulted in discarding the corresponding ectopic beat, therefore increasing the apparent VV value of the following beat after the compensatory pause. However, this was not a practical issue here, since we were still able to detect the longer-than-normal following VV; this could potentially be a limitation if we use this classification approach for the purpose of automated separate image reconstruction according to the type of beat (i.e., normal beat following a normal beat, abnormal beat following a normal beat, normal beat following an abnormal beat). Finally, although our automated arrhythmia detection and sorting algorithm is promising, it is still not completely accurate for the classification of AF and PVC; this performance also needs to be further evaluated in a larger scale study.

## Conclusions

In conclusion, since arrhythmia can lead to reduced quality of CMR, its detection and classification, and its incorporation in the imaging process, are potentially very important. Our study shows that we can use an automated approach to reliably detect arrhythmic patterns and sort AF from PVC, using a motion-related signal in self-gated cine CMR sequences, such as XD-GRASP, therefore offering the possibility of developing better automatic detection and sorting algorithms for arrhythmia in such CMR examinations. This approach is also likely to be useful for similar detection and classification of arrhythmias using the conventional ECG signal acquired during CMR.

## Abbreviations

A: Arrhythmic patients
AF: Atrial fibrillation
AIC: Akaike information criterion
AUC: Area under the curve
BIC: Bayesian information criterion
CMR: Cardiovascular Magnetic Resonance
CT: Computed tomography
ECG: Electrocardiogram
meanVV: Weighted mean of valley intervals
medVV: Median of valley intervals
NA: Non arrhythmic patients
NLLH: Negative Log Likelihood Function
PVC: Premature Ventricular Contraction
ROC: Receiver Operating Characteristic
SAX: Short axis
SSFP: Steady-state free precession
V_i_: Ith valley of the cardiac motion-related signal
VV: Valley intervals
VVHR: Half range
VVTR: Total range
VVval: Number of values of valley intervals
XD-GRASP: EXtra Dimension-Golden angle Radial Sparse Parallel
